# Side effects of low-dose tamoxifen: results from a six-armed randomised controlled trial in healthy women

**DOI:** 10.1038/s41416-023-02293-z

**Published:** 2023-05-06

**Authors:** Mattias Hammarström, Marike Gabrielson, Alessio Crippa, Andrea Discacciati, Martin Eklund, Cecilia Lundholm, Magnus Bäcklund, Yvonne Wengström, Signe Borgquist, Jenny Bergqvist, Mikael Eriksson, José Tapia, Kamila Czene, Per Hall

**Affiliations:** 1 Department of Medical Epidemiology and Biostatistics, Karolinska Institutet, Stockholm, Sweden; 2 Department of Neurobiology, Care Science and Society, Division of Nursing and Theme Cancer, Karolinska University Hospital, Stockholm, Sweden; 3 Department of Oncology, Aarhus University Hospital and Aarhus University, Aarhus, Denmark; 4 Department of Clinical Sciences Lund, Oncology, Lund University and Skåne University Hospital, Lund, Sweden; 5 Department of Oncology, Capio St Görans Hospital, Stockholm, Sweden; 6 Department of Oncology, Södersjukhuset, Stockholm, Sweden

**Keywords:** Breast cancer, Cancer therapy, Cancer prevention

## Abstract

**Background:**

Adherence to adjuvant tamoxifen therapy is suboptimal, and acceptance of tamoxifen for primary prevention is poor. Published results indicate effect of low-dose tamoxifen therapy. Using questionnaire data from a randomised controlled trial, we describe side effects of standard and low-dose tamoxifen in healthy women.

**Methods:**

In the KARISMA trial, 1440 healthy women were randomised to 6 months of daily intake of 20, 10, 5, 2.5, 1 mg of tamoxifen or placebo. Participants completed a 48-item, five-graded Likert score symptom questionnaire at baseline and follow-up. Linear regression models were used to identify significant changes in severity levels across doses and by menopausal status.

**Results:**

Out of 48 predefined symptoms, five were associated with tamoxifen exposure (hot flashes, night sweats, cold sweats, vaginal discharge and muscle cramps). When comparing these side effects in premenopausal women randomised to low doses (2.5, 5 mg) versus high doses (10, 20 mg), the mean change was 34% lower in the low-dose group. No dose-dependent difference was seen in postmenopausal women.

**Conclusions:**

Symptoms related to tamoxifen therapy are influenced by menopausal status. Low-dose tamoxifen, in contrast to high-dose, was associated with less pronounced side effects, a finding restricted to premenopausal women. Our findings give new insights which may influence future dosing strategies of tamoxifen in both the adjuvant and preventive settings.

**Trial registration:**

ClinicalTrials.gov ID: NCT03346200.

## Introduction

Adjuvant tamoxifen treatment reduces recurrence and death from oestrogen receptor (ER)-positive breast cancer in pre- and postmenopausal women [1–3]. Furthermore, tamoxifen is approved for the primary prevention of breast cancer in both the US and UK. However, uptake is low; more than 90% of high-risk women do not comply with preventive treatment with anti-oestrogens. One of several suggested explanations for the low uptake is concerns of side effects [4–8].

Tamoxifen is a selective ER modulator that acts as an oestrogen antagonist in the breast, along with oestrogen-like agonistic effects on the endometrium, skeleton, coagulation system, and metabolism of lipids [2, 9, 10]. Vasomotor and vaginal symptoms are well-established side effects of tamoxifen [9, 11]. Serious, but uncommon, side effects are venous thromboembolic events and endometrial cancer, the latter only seen in postmenopausal women [2, 11, 12].

Most studies on tamoxifen side effects have been restricted to breast cancer patients making it difficult to disentangle tamoxifen-specific side effects [2]. The National Surgical Adjuvant Breast and Bowel Project (NSABP P-1) is the largest of four prevention trials [13–16] including 13,388 pre- and postmenopausal women randomised to either placebo or 20 mg tamoxifen. Hot flashes, vaginal discharge and irregular menses were the most common side effects reported [13].

Side effects not only affect adherence to adjuvant tamoxifen treatment [17–20] but also reduce the willingness to prescribe tamoxifen to healthy women at increased risk of breast cancer [5, 21]. In the primary prevention trial IBIS-1, the women taking 20 mg tamoxifen demonstrated higher dropout rates from first 12 months and inferior long-term adherence compared to the placebo group [8]. Lower doses of tamoxifen have been discussed over the years as a strategy to increase adherence to preventive and adjuvant therapy. The randomised trial TAM-01 indicate that 5 mg tamoxifen daily for 3 years could be a sufficient therapeutic dose for risk reduction in high-risk women with a 50% reduction of invasive and non-invasive breast cancer events [22]. A recent 10-year follow-up demonstrates a durable preventive effect [23].

We recently published the first results of the KARISMA trial [24]. We tested if lower doses of tamoxifen reduce mammographic density and found that 2.5, 5 and 10 mg of tamoxifen were as effective as 20 mg, a finding restricted to premenopausal women. All women (including both pre- and postmenopausal) randomised to 2.5 mg of tamoxifen reported significantly fewer severe vasomotor symptoms compared to the 20 mg arm, 20.5% (95% CI:15.5–26.6) compared to 34.0% (95% CI: 27.8–40.7), respectively. In this exploratory analysis, we took advantage of the full 48-item KARISMA symptom questionnaire to describe the complete side effect spectra at different doses of tamoxifen in healthy women. The aim was to study how questionnaire-reported side effects are associated with low and high doses of tamoxifen, and the influence of menopausal status.

## Materials and methods

### Study design and participants

This is an exploratory analysis of the secondary objective in the KARISMA trial (assess the level of side effects at lower doses and compare to the 20 mg arm), the analyses are thereby not predefined in the protocol. The full study protocol can be found elsewhere [24].

The KARISMA trial (EudraCT: 2016-000882-22) is an investigator-initiated, double-blind, randomised placebo-controlled six-armed dose-determination trial conducted in Sweden between 2016 and 2019 [24]. The primary outcome is the identification of the minimal dose of tamoxifen non-inferior in its ability to reduce mammographic density compared to 20 mg of tamoxifen. Healthy Swedish women, age 40–74 years, attending the population-based national mammography screening programme, were invited. Main exclusion criteria were history of cardiovascular disease, high blood pressure, hormonal replacement therapy, oral contraceptives, concomitant medication interfering with CYP2D6 expression, and low mammographic density, corresponding to a BI-RADS A score [25]. Postmenopausal status was defined as absence of bleedings during last 12 months. Participants were treated daily for 6 months and randomised into six arms: placebo, 1, 2.5, 5, 10 or 20 mg of tamoxifen.

### Questionnaire data

The KARISMA symptom questionnaire consist of 48 predefined questions including side effects associated to endocrine treatment and more general symptoms potentially affecting the quality of life (Supplementary Methods). Nineteen questions were selected from the validated Functional Assessment of Cancer Therapy - Endocrine Symptoms (FACT-ES Additional Concerns) questionnaire [26]. Four questions were identified in the Summary of Product Characteristics (SmPC) of Tamoxifen (Mylan) [12] and three symptoms were found in the literature or anecdotally to be associated to tamoxifen treatment [27, 28]. The remaining 22 questions addressed general common symptoms related to cancer treatment and were derived from the Memorial Symptom Assessment Scale (MSAS) [29, 30] (Supplementary Methods).

The instruction for the 26 endocrine-treatment-related questions was “Mark every symptom as it applies to the last 30 days”, whilst for the 22 general symptoms the instruction read “How much did the symptom bother or distress you during the last 30 days”. The MSAS questionnaire also included the dimensions frequency and severity, but those were not included in this analyse since the answer options are not consistent with FACT-ES. For all 48 symptoms, the five-graded Likert scale [31] was used for self-assessment with the answer options: 0 = ‘not at all’, 1 = ‘a little bit’, 2 = ‘somewhat’, 3 = ‘quite a bit’, and 4 = ‘very much’.

Participants answered the web questionnaire at inclusion, at 1-, 3- and 6 months following therapy initiation. Women who discontinued treatment were asked to complete a questionnaire at the date of discontinuation. Participant who did not complete the questionnaire within 2 weeks, received a reminder via phone. Answers from the first and last questionnaire were used in the analyses. Missing answers in the questionnaire were excluded from the analyses.

### Anthropometric measures

Measurements of weight, length and waist circumference were performed at baseline and at end of treatment. The measurements were assessed from calibrated scales and performed by research nurses at the study centre.

### Identification of tamoxifen-associated symptoms and analysis of dose dependency

Change in reported symptom severity from baseline to end of treatment was calculated by subtracting the baseline Likert-scale score (0–4) from the score reported at the end of study (6 months or date of discontinuation). A mean of the individual change was calculated for each of the 48 predefined symptoms. The rationale for this method was to take background conditions into consideration, for comparison of pre- and postmenopausal women, and to capture the magnitude of change. To recognise side effects associated with tamoxifen, the placebo group was contrasted to the 20 mg group and symptoms significantly related to tamoxifen were identified.

In the dose-dependent analyses, we contrasted the mean change of the score for those symptoms significantly associated with tamoxifen. In a subsequent analysis, we contrasted low-dose tamoxifen, defined as 2.5 and 5 mg, to high-dose tamoxifen (10 and 20 mg). The reason not to include the 1 mg tamoxifen dose in the low-dose group was that no RCTs, including our own [22, 24, 32], has shown an effect of 1 mg.

### Statistical analysis

Linear regression models were used for contrasting change in mean scores for the 20 mg dose arm versus the placebo arm to identify symptoms and anthropometric measures associated with the standard dose of tamoxifen. Similar regression models were employed in the dose dependency analyses, where we estimated the association between change in mean scores and dose arms, limited to the symptoms identified to be significantly associated with tamoxifen. In contrast to the analysis where tamoxifen-associated symptoms were identified using placebo as a reference, we presented the dose-dependent results using the 20 mg group for comparison, to test how intensity was affected by lower doses compared to standard dose treatment in a clinical setting. Every dose was contrasted to the 20 mg arm and in addition the association between higher dose and severity change was tested using linear regression analysis with actual dose (0, 1, 2.5, 5, 10 and 20 mg) as independent variable.

We further computed a score of the identified top five symptoms by summing means of Likert score change from baseline to end of treatment. Linear regression was used to contrast low-dose vs high-dose tamoxifen stratified by menopausal status. In the same manner, increase in Likert scores was calculated and categorised to ≥1, ≥2, ≥3, ≥4 and ≥5 Likert score. Logistic regression was used to compare differences in proportion of women increasing by categories, stratified by menopausal status and tamoxifen dose.

All calculations were performed on the per protocol population and stratified on menopausal status. Wald tests were performed for testing associations and possible interactions by menopausal status. All *P* values were two-sided and confidence intervals (CI) were set at the 95% level. *P* values and 95% confidence intervals have not been adjusted for multiplicity and should therefore be interpreted with caution. Analyses were conducted in R version 4.1 and SPSS version 28.

## Results

### Baseline characteristics, adherence to treatment and completeness of questionnaires

**Fig. 1:**
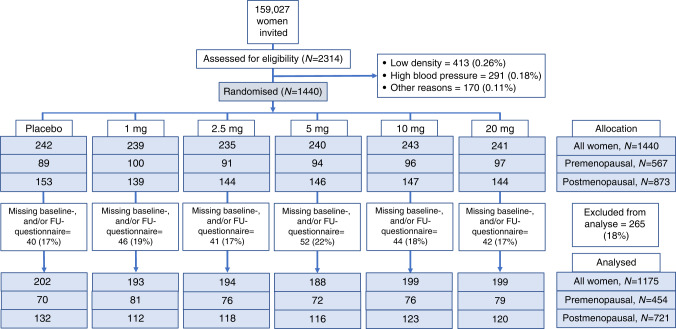
CONSORT diagram. FU follow-up.

A total of 159,027 women were invited and 1440 women were included in the trial which corresponds to a recruitment rate of ~1% (Fig. 1). Of the 1440 women included, 1175 (82%) participants completed the baseline and follow-up questionnaire, either after 6 months of medication (*n* = 1010 (70%)), or at the date of discontinuation (*n* = 165 (11%)) (Fig. 1). A completed questionnaire was defined as at least 90% of the questions were answered at baseline and at the end of study. The average completeness for all 48 questions was 98%. Seven questions had a completeness below the average whereof the questions with lowest response rate were 'pain at intercourse' and 'lost interest in sex' (68% and 81%, respectively). The 1175 women, whereof 454 (39%) premenopausal and 721 (61%) postmenopausal, constitute the per protocol population and form the basis of our study (Fig. 1 and Table 1).

**Table 1:** Baseline characteristics and adherence to treatment of the included 1175 women, stratified by tamoxifen dose.

		Tamoxifen dose in mg					
Characteristic	All	0	1	2.5	5	10	20
Number of women	1175	202	193	194	188	199	199
Mean age at baseline, years (SD)	55.7 (9.7)	56.4 (9.7)	54.5 (9.5)	55.3 (9.9)	55.5 (9.9)	56.5 (10.0)	55.9 (9.3)
Number of premenopausal women (%)	454 (38.6)	70 (34.7)	81 (42.0)	76 (39.2)	72 (38.3)	76 (38.2)	79 (39.7)
Number of postmenopausal women (%)	721 (61.4)	132 (65.3)	112 (58.0)	118 (60.8)	116 (61.7)	123 (61.8)	120 (60.3)
Mean age at menopause, years (SD)	50.0 (5.12)	50.0 (4.78)	50.0 (5.11)	50.3 (5.66)	49.6 (5.69)	49.3 (4.61)	50.4 (4.97)
Mean age at menarche, years (SD)	13.0 (1.36)	13.2 (1.40)	13.0 (1.39)	12.8 (1.37)	13.0 (1.32)	13.1 (1.37)	12.9 (1.32)
Mean BMI (SD)	25.4 (3.8)	25.0 (3.6)	25.5 (3.8)	25.8 (4.3)	25.6 (3.9)	25.4 (3.7)	25.4 (3.3)
Mean number of pregnancies (SD)	2.6 (1.6)	2.5 (1.4)	2.6 (1.6)	2.8 (1.6)	2.4 (1.6)	2.5 (1.5)	2.6 (1.7)
Mean age at first birth, years (SD)	28.8 (5.4)	28.7 (5.5)	28.8 (5.3)	29.0 (5.3)	28.7 (5.5)	28.8 (5.5)	28.9 (5.5)
Number of current smokers (%)	135 (12)	17 (8)	22 (11)	18 (9)	19 (10)	26 (13)	33 (17)
Mean gram/week of alcohol last year (SD)	49.4 (61.4)	53.6 (64.7)	48.9 (59.1)	43.3 (43.9)	49.4 (60.8)	53.7 (81.8)	47.5 (51.6)
*Adherence*							
Compliant to treatment^a^ (%)							
All	996 (85.3)	182 (90.1)	170 (88.5)	159 (82.0)	152 (82.2)	164 (83.2)	169 (85.8)
Premenopausal	393 (87.1)	62 (88.6)	71 (87.7)	64 (84.2)	64 (91.4)	65 (85.5)	67 (85.9)
Postmenopausal	603 (84.2)	120 (90.9)	99 (89.2)	95 (80.5)	88 (76.5)	99 (81.8)	102 (85.7)
Number of women that discontinued treatment due to side effects (%)							
All	132 (11.2)	13 (6.4)	15 (7.8)	28 (14.4)	27 (14.4)	27 (13.6)	22 (11.1)
Premenopausal	35 (7.7)	4 (5.7)	6 (7.4)	6 (7.9)	5 (6.9)	7 (9.2)	7 (8.9)
Postmenopausal	97 (13.5)	9 (6.8)	9 (8.0)	22 (18.6)	22 (19.0)	20 (16.3)	15 (12.5)

*BMI* body mass index, *SD* standard deviation.

^a^Compliant to treatment: defined per protocol as 80 % of tablets taken. Missing information for 8 participants (3 premenopausal, 5 postmenopausal).

Overall, 996 (85.3%) of the participants were compliant to treatment (measured as 80% of tablets taken) (Table 1). Discontinuation due to side effects was seen in 132 (11.2 %) participants. The difference in discontinuation between premenopausal (7.7%) and postmenopausal (13.5%) women was significant (*P* = 0.002) (Table 1).

There were no major differences in baseline characteristics between tamoxifen dose groups, with two exceptions. Smoking was more prevalent in the 20 mg arm compared to the placebo arm and 5 mg arm (Table 1), and the mean Likert score of hot flashes was higher in the 20 mg arm compared to lower doses (Supplementary Table 1).

### Identification of side effects associated with tamoxifen and influence of menopausal status on tamoxifen-associated side effects

Five symptoms (‘hot flashes’, ‘cold sweats’, ‘night sweats’, ‘vaginal discharge’ and ‘muscle cramps’) were found to have a significant change from start to the end of treatment in both pre- and postmenopausal women when contrasting the effect of 20 mg tamoxifen compared to women on placebo. These symptoms also demonstrated the largest magnitude in mean change of Likert score (Table 2).

**Table 2:** The individual change in Likert score in 48-items of the KARISMA symptom questionnaire from baseline to end of treatment (at 6 months or date of discontinuation), contrasting the placebo to the group treated with 20 mg of tamoxifen, for all women combined and stratified by menopausal status.

	All (*N* = 401)		Premenopausal (*n* = 149)		Postmenopausal (*n* = 252)		Interaction menopausal status
Group symptom	Mean change 20 to 0 mg (ref.)^a^	95% confidence interval	Mean change 20 to 0 mg (ref.)^a^	95% confidence interval	Mean change 20 to 0 mg (ref.)^a^	95% confidence interval	*P* value^b^
*Vasomotor*							
Hot flashes	**0.39**	[**0.19**, **0.59**]	**0.44**	[**0.10**, **0.79**]	**0.36**	[**0.11**, **0.60**]	0.694
Cold sweats	**0.35**	[**0.20**, **0.50**]	**0.30**	[**0.06**, **0.53**]	**0.38**	[**0.19**, **0.58**]	0.575
Night sweats	**0.45**	[**0.24**, **0.67**]	**0.55**	[**0.16**, **0.95**]	**0.39**	[**0.14**, **0.64**]	0.503
*Gynaecological/breast*							
Vaginal discharge	**0.51**	[**0.35**, **0.68**]	**0.41**	[**0.10**, **0.72**]	**0.60**	[**0.41**, **0.78**]	0.301
Vaginal itching/irritation	**0.28**	[**0.12**, **0.43**]	0.26	[−0.01, 0.53]	**0.29**	[**0.09**, **0.49**]	0.850
Vaginal bleeding or spotting	−0.07	[−0.16, 0.02]	−0.09	[−0.32, 0.14]	−0.05	[−0.12, 0.01]	0.785
Vaginal dryness	−0.04	[−0.23, 0.15]	0.18	[−0.07, 0.43]	−0.19	[−0.45, 0.08]	0.050
Problems with urination	0.06	[−0.01, 0.12]	0.06	[−0.08, 0.20]	0.05	[−0.02, 0.12]	0.885
Sensitive/tender breasts	−0.04	[−0.19, 0.10]	**−0.28**	[**−0.56**, **−0.01**]	0.11	[−0.05, 0.27]	**0.014**
*Sexual*							
Pain or discomfort with intercourse	0.15	[−0.04, 0.33]	0.14	[−0.07, 0.35]	0.14	[−0.13, 0.42]	0.991
Lost interest in sex	0.07	[−0.18, 0.33]	−0.33	[−0.72, 0.07]	**0.34**	[**0.01**, **0.67**]	**0.011**
*Musculoskeletal*							
Joint pain	−0.05	[−0.26, 0.15]	−0.13	[−0.43, 0.16]	0.00	[−0.27, 0.27]	0.518
Muscle cramps	**0.66**	[**0.47**, **0.86**]	**0.41**	[**0.12**, **0.70**]	**0.83**	[**0.57**, **1.08**]	**0.034**
Numbness in hands/ feet	0.01	[−0.11, 0.13]	−0.09	[−0.27, 0.09]	0.08	[−0.08, 0.23]	0.164
*Skin/subcutaneous/mucous*							
Fragile mucous membranes	0.05	[−0.12, 0.22]	0.12	[−0.12, 0.36]	0.00	[−0.24, 0.23]	0.465
Dry mouth	**0.12**	[**0.01**, **0.22**]	0.15	[−0.03, 0.33]	0.09	[−0.05, 0.23]	0.608
Skin rashes	**0.16**	[**0.02**, **0.30**]	**0.30**	[**0.04**, **0.56**]	0.08	[−0.08, 0.24]	0.166
Itching	**0.18**	[**0.05**, **0.32**]	0.22	[−0.02, 0.47]	0.16	[−0.01, 0.33]	0.674
Hair loss	−0.05	[−0.21, 0.10]	−0.18	[−0.39, 0.04]	0.03	[−0.19, 0.25]	0.183
*Metabolism/nutrition*							
Gained weight	−0.02	[−0.22, 0.18]	−0.11	[−0.44, 0.22]	0.04	[−0.20, 0.28]	0.475
Feeling bloated	0.04	[−0.15, 0.22]	−0.04	[−0.37, 0.29]	0.10	[−0.12, 0.31]	0.496
Swollen arms/legs	0.04	[−0.03, 0.11]	0.07	[−0.04, 0.19]	0.02	[−0.06, 0.10]	0.436
Abdominal obesity	−0.01	[−0.17, 0.15]	−0.01	[−0.26, 0.24]	−0.01	[−0.23, 0.20]	0.989
*Gastrointestinal*							
Diarrhoea	**−0.16**	[**−0.28**, **−0.03**]	−0.21	[−0.43, 0.01]	−0.13	[−0.28, 0.03]	0.558
Constipation	−0.03	[−0.16, 0.10]	−0.11	[−0.33, 0.12]	0.02	[−0.14, 0.18]	0.373
Nausea	−0.06	[−0.18, 0.06]	−0.23	[−0.47, 0.02]	0.04	[−0.09, 0.16]	0.063
Vomiting	−0.07	[−0.15, 0.01]	−0.07	[−0.23, 0.09]	−0.08	[−0.15, 0.00]	0.956
Lack of appetite	**0.05**	[**0.00**, **0.09**]	0.08	[0.00, 0.16]	0.03	[−0.03, 0.08]	0.296
Change in the way food taste	0.01	[−0.02, 0.04]	0.01	[−0.01, 0.04]	0.01	[−0.04, 0.06]	0.918
Difficulty swallowing	0.00	[−0.06, 0.06]	0.00	[0.00, 0.00]	0.00	[−0.09, 0.10]	0.975
*Psychological*							
Mood swings	0.05	[−0.12, 0.22]	0.00	[−0.30, 0.30]	0.10	[−0.10, 0.30]	0.586
Feeling irritable	0.07	[−0.10, 0.24]	−0.02	[−0.31, 0.26]	0.13	[−0.08, 0.35]	0.381
Feeling nervous	**−0.13**	[**−0.23**, **−0.02**]	−0.17	[−0.36, 0.02]	−0.09	[−0.21, 0.03]	0.466
Feeling sad	−0.12	[−0.28, 0.04]	−0.25	[−0.60, 0.09]	−0.03	[−0.19, 0.12]	0.265
Worrying	0.00	[−0.16, 0.16]	−0.01	[−0.31, 0.29]	0.02	[−0.16, 0.21]	0.855
Lack of energy	−0.07	[−0.25, 0.10]	−0.09	[−0.38, 0.20]	−0.04	[−0.26, 0.17]	0.786
Difficulty concentrating	−0.07	[−0.19, 0.06]	−0.07	[−0.30, 0.16]	−0.05	[−0.20, 0.09]	0.900
Sleeping difficulties	−0.16	[−0.39, 0.08]	−0.12	[−0.50, 0.27]	−0.18	[−0.48, 0.11]	0.780
Feeling drowsy	0.02	[−0.08, 0.11]	−0.04	[−0.21, 0.14]	0.05	[−0.05, 0.16]	0.404
“I don’t look like myself”	0.00	[−0.14, 0.13]	−0.07	[−0.29, 0.15]	0.04	[−0.13, 0.21]	0.445
*Nervous system*							
Pain	−0.18	[−0.40, 0.05]	−0.39	[−0.79, 0.01]	−0.06	[−0.33, 0.21]	0.177
Dizziness	0.04	[−0.11, 0.18]	−0.04	[−0.28, 0.20]	0.09	[−0.08, 0.27]	0.366
Headache	0.08	[−0.11, 0.27]	0.21	[−0.16, 0.57]	0.01	[−0.20, 0.22]	0.365
Heart palpitations	0.03	[−0.09, 0.14]	0.09	[−0.10, 0.27]	−0.01	[−0.16, 0.15]	0.455
*Respiratory*							
Cough	0.01	[−0.15, 0.18]	−0.10	[−0.33, 0.14]	0.08	[−0.15, 0.31]	0.279
Shortness of breath	0.07	[−0.04, 0.18]	0.13	[−0.04, 0.31]	0.04	[−0.11, 0.18]	0.404
*Eyes*							
Sight/eye changes	0.06	[−0.14, 0.26]	0.01	[−0.30, 0.33]	0.08	[−0.17, 0.33]	0.746
Dry eyes	0.06	[−0.11, 0.24]	0.09	[−0.19, 0.37]	0.05	[−0.18, 0.27]	0.801

^a^Difference in mean change comparing 20 to 0 mg (ref.) tamoxifen at end of treatment (at 6 months or date of discontinuation).

^b^*P* values for interaction of menopausal status on the effect of tamoxifen.

Significant values are in bold (*P* < 0.05).

‘Skin rashes’ and a negative mean change in ‘sensitive/tender breasts’ showed a significant association with tamoxifen in premenopausal women only while ‘vaginal itching/irritation’ and ‘lost interest in sex’ were significant in postmenopausal women (Table 2). Both pre- and postmenopausal women in the tamoxifen arm reported problems with muscle cramps. However, postmenopausal women had significantly higher differences from start to end compared to premenopausal, with a doubled magnitude of mean change of severity score. Premenopausal women reported higher severity of vaginal dryness in contrast to postmenopausal women (Table 2).

Premenopausal women treated with 20 mg tamoxifen experienced significantly reduced waist circumference, weight and BMI (Table 3). No significant changes in anthropometric measures related to tamoxifen were seen in postmenopausal women.

**Table 3:** Mean individual change of three anthropometric measures, from start to end of treatment (exit or 6 months), comparing 20 mg group to the placebo group (reference).

	All (*N* = 401)		Premenopausal (*n* = 149)		Postmenopausal (*n* = 252)		Interaction menopausal status
Anthropometrics	Mean change 20 to 0 mg (ref.)^a^	95% confidence interval	Mean change 20 to 0 mg (ref.)^a^	95% confidence interval	Mean change 20 to 0 mg (ref.)^a^	95% confidence interval	*P* value^b^
BMI (kg/m^2^)	**−0.22**	[**−0.37**, **−0.06**]	**−0.28**	[**−0.56**, **−0.01**]	−0.17	[−0.35, 0.02]	0.505
Weight (kg)	**−0.59**	[**−1.01**, **−0.16**]	**−0.80**	[**−1.55**, **−0.05**]	−0.43	[−0.92, 0.07]	0.417
Waist circumference (cm)	−0.74	[−2.57, 1.08]	**−3.53**	[**−6.73**, **−0.32**]	0.94	[−1.24, 3.12]	**0.024**

*BMI* body mass index.

^a^Difference in mean change comparing 20 to 0 mg (ref.) tamoxifen at end of treatment (at 6 months or date of discontinuation).

^b^*P* values for interaction of menopausal status on the effect of tamoxifen.

Significant values are in bold (*P* < 0.05).

### Dose-dependent side effects

Among the five identified symptoms associated with 20 mg tamoxifen in both pre- and postmenopausal women, the change in severity tended to be lower at lower doses. Consequently, all five symptoms demonstrated a linear association with tamoxifen dose when analysing pre- and postmenopausal women combined (‘hot flashes’ *P* = 0.002; ‘night sweats’ *P* = 0.003; ‘cold sweats’ *P* = <0.001; ‘vaginal discharge’ *P* = 0.002; ‘muscle cramps’ *P* = <0.001) (Table 4). Association between dose and severity change was also seen in ‘vaginal itching/irritation’ (*P* = 0.021); ‘diarrhoea’ (*P* = 0.005); ‘itching’ (*P* = 0.033) (Table 4), as well as in the anthropometric measures ‘weight’ (*P* = 0.005) and ‘BMI’ (*P* = 0.004) (Supplementary Table 2). For premenopausal women only, significant association for severity change with lower doses was seen for ‘skin rashes’ and ‘feeling nervous’ (*P* = 0.047 and *P* = 0.040, respectively) (Table 4). The symptom ‘sensitive/tender breasts’ demonstrated a linear association of less severity by dose in postmenopausal women only (*P* = 0.021) (Table 4).

**Table 4:** The individual change in Likert score from baseline to end of treatment (at 6 months or date of discontinuation), comparing all doses, including placebo to the 20 mg tamoxifen arm, for all women combined and stratified by menopausal status.

		Tamoxifen dose in mg						
Symptom	*N*	0	1	2.5	5	10	20	Association with dose
		Mean change (95% CI)^a^	Mean change (95% CI)^a^	Mean change (95% CI)^a^	Mean change (95% CI)^a^	Mean change (95% CI)^a^	Ref.	*P* value^b^
*Vasomotor*								
Hot flashes								
All	1161	**−0.39** (**−0.59**, **−0.18**)	−0.20 (−0.40, 0.01)	0.02 (−0.18, 0.23)	0.08 (−0.13, 0.29)	0.17 (−0.03, 0.38)	Ref.	**0.002**
Premenopausal	446	**−0.44** (**−0.77**, **−0.11**)	**−0.34** (**−0.66**, **−0.02**)	−0.11 (−0.43, 0.21)	−0.06 (−0.39, 0.27)	0.26 (−0.06, 0.58)	Ref.	**0.002**
Postmenopausal	715	**−0.36** (**−0.62**, **−0.10**)	−0.09 (−0.36, 0.18)	0.11 (−0.16, 0.38)	0.17 (−0.10, 0.44)	0.12 (−0.14, 0.39)	Ref.	0.097
Night sweats								
All	1166	**−0.45** (**−0.67**, **−0.24**)	−0.10 (−0.32, 0.11)	−0.08 (−0.30, 0.13)	0.09 (−0.13, 0.31)	0.06 (−0.16, 0.27)	Ref.	**0.003**
Premenopausal	450	**−0.55** (**−0.92**, **−0.18**)	−0.23 (−0.58, 0.13)	−0.18 (−0.54, 0.18)	−0.10 (−0.46, 0.27)	0.08 (−0.28, 0.44)	Ref.	**0.010**
Postmenopausal	716	**−0.39** (**−0.65**, **−0.13**)	−0.02 (−0.29, 0.25)	−0.01 (−0.28, 0.25)	0.21 (−0.06, 0.48)	0.05 (−0.21, 0.32)	Ref.	0.090
Cold sweats								
All	1162	**−0.35** (**−0.51**, **−0.19**)	**−0.27** (**−0.43**, **−0.10**)	−0.15 (−0.31, 0.01)	−0.10 (−0.26, 0.06)	−0.12 (−0.28, 0.04)	Ref.	**<0.001**
Premenopausal	448	**−0.30** (**−0.52**, **−0.07**)	**−0.31** (**−0.53**, **−0.09**)	−0.14 (−0.36, 0.08)	−0.20 (−0.42, 0.03)	0.02 (−0.2, 0.24)	Ref.	**0.001**
Postmenopausal	714	**−0.38** (**−0.6**, **−0.17**)	**−0.23** (**−0.45**, **0.00**)	−0.16 (−0.38, 0.06)	−0.04 (−0.26, 0.18)	−0.21 (−0.43, 0.01)	Ref.	**0.006**
*Gynaecological/breast*								
Vaginal discharge								
All	1167	**−0.51** (**−0.71**, **−0.31**)	−0.06 (−0.26, 0.14)	0.15 (−0.05, 0.35)	−0.10 (−0.30, 0.10)	0.20 (−0.01, 0.40)	Ref.	**0.002**
Premenopausal	450	**−0.41** (**−0.73**, **−0.09**)	−0.04 (−0.35, 0.27)	0.10 (−0.22, 0.41)	**−0.35** (**−0.66**, **−0.03**)	0.05 (−0.27, 0.36)	Ref.	0.131
Postmenopausal	717	**−0.60** (**−0.84**, **−0.35**)	−0.06 (−0.32, 0.19)	0.19 (−0.06, 0.44)	0.05 (−0.20, 0.30)	**0.28** (**0.03**, **0.53**)	Ref.	**0.003**
Vaginal itching/irritation								
All	1164	**−0.28** (**−0.43**, **−0.12**)	−0.06 (−0.22, 0.1)	−0.06 (−0.21, 0.1)	−0.09 (−0.25, 0.07)	−0.06 (−0.22, 0.09)	Ref.	**0.021**
Premenopausal	452	−0.26 (−0.53, 0.01)	−0.01 (−0.27, 0.25)	0.03 (−0.23, 0.30)	−0.06 (−0.33, 0.20)	−0.07 (−0.33, 0.19)	Ref.	0.412
Postmenopausal	719	**−0.29** (**−0.48**, **−0.10**)	−0.09 (−0.29, 0.11)	−0.11 (−0.31, 0.09)	−0.10 (−0.30, 0.10)	−0.06 (−0.26, 0.14)	Ref.	**0.020**
Sensitive/tender breasts								
All	1172	0.04 (−0.09, 0.17)	−0.06 (−0.19, 0.07)	−0.12 (−0.25, 0.01)	−0.05 (−0.18, 0.08)	−0.09 (−0.22, 0.04)	Ref.	0.725
Premenopausal	452	**0.28** (**0.02**, **0.54**)	0.16 (−0.09, 0.42)	−0.05 (−0.30, 0.21)	0.06 (−0.20, 0.32)	0.11 (−0.15, 0.36)	Ref.	0.170
Postmenopausal	720	−0.11 (−0.25, 0.02)	**−0.21** (**−0.35**, **−0.07**)	**−0.18** (**−0.31**, **−0.04**)	−0.12 (−0.26, 0.01)	**−0.22** (**−0.35**, **−0.08**)	Ref.	**0.021**
*Sexual*								
Lost interest in sex								
All	947	−0.07 (−0.34, 0.19)	−0.10 (−0.36, 0.16)	0.11 (−0.15, 0.38)	−0.09 (−0.36, 0.17)	0.12 (−0.14, 0.38)	Ref.	0.476
Premenopausal	412	0.33 (−0.07, 0.72)	0.00 (−0.39, 0.39)	0.39 (−0.01, 0.78)	0.10 (−0.29, 0.50)	0.34 (−0.05, 0.72)	Ref.	0.319
Postmenopausal	535	**−0.34** (**−0.68**, **0.00**)	−0.18 (−0.53, 0.17)	−0.09 (−0.43, 0.26)	−0.26 (−0.62, 0.10)	−0.07 (−0.42, 0.29)	Ref.	0.087
*Musculoskeletal*								
Muscle cramps								
All	1169	**−0.66** (**−0.85, −0.48**)	**−0.44** (**−0.63**, **−0.25**)	**−0.26** (**−0.45, −0.07**)	−0.17 (−0.36, 0.02)	−0.04 (−0.22, 0.15)	Ref.	**<0.001**
Premenopausal	450	**−0.41** (**−0.68**, **−0.14**)	**−0.28** (**−0.54**, **−0.02**)	−0.09 (−0.35, 0.17)	−0.16 (−0.42, 0.11)	0.12 (−0.14, 0.38)	Ref.	**0.002**
Postmenopausal	719	**−0.83** (**−1.07**, **−0.58**)	**−0.54** (**−0.8**, **−0.29**)	**−0.37** (**−0.63, −0.12**)	−0.19 (−0.45, 0.07)	−0.14 (−0.4, 0.11)	Ref.	**<0.001**
*Skin/subcutaneous/mucous*								
Dry mouth								
All	1175	−0.12 (−0.23, 0)	−0.08 (−0.2, 0.03)	0.04 (−0.07, 0.16)	0.02 (−0.1, 0.14)	−0.05 (−0.17, 0.07)	Ref.	0.250
Premenopausal	454	−0.15 (−0.31, 0.01)	−0.12 (−0.27, 0.04)	−0.06 (−0.22, 0.1)	−0.04 (−0.2, 0.12)	**−0.18** (**−0.33, −0.02**)	Ref.	0.195
Postmenopausal	721	−0.09 (−0.25, 0.07)	−0.07 (−0.23, 0.1)	0.11 (−0.05, 0.28)	0.06 (−0.1, 0.23)	0.03 (−0.13, 0.19)	Ref.	0.575
Skin rashes								
All	1156	**−0.16** (**−0.3**, **−0.01**)	−0.14 (−0.29, 0.01)	−0.09 (−0.24, 0.05)	−0.06 (−0.21, 0.09)	**−0.15** (**−0.29**, **0.00**)	Ref.	0.058
Premenopausal	446	**−0.30** (**−0.52**, **−0.07**)	−0.14 (−0.36, 0.08)	−0.08 (−0.30, 0.14)	0.00 (−0.22, 0.23)	−0.07 (−0.29, 0.15)	Ref.	**0.047**
Postmenopausal	710	−0.08 (−0.27, 0.11)	−0.14 (−0.34, 0.06)	−0.10 (−0.30, 0.09)	−0.09 (−0.29, 0.10)	**−0.20** (**−0.39**, **0.00**)	Ref.	0.369
Itching								
All	1175	**−0.18** (**−0.32**, **−0.05**)	−0.11 (−0.25, 0.03)	−0.02 (−0.16, 0.12)	−0.05 (−0.19, 0.09)	−0.04 (−0.18, 0.10)	Ref.	**0.033**
Premenopausal	454	−0.22 (−0.45, 0.00)	−0.15 (−0.37, 0.07)	−0.11 (−0.34, 0.11)	0.00 (−0.23, 0.23)	−0.10 (−0.32, 0.12)	Ref.	0.090
Postmenopausal	721	−0.16 (−0.33, 0.01)	−0.08 (−0.26, 0.10)	0.04 (−0.13, 0.22)	−0.07 (−0.25, 0.10)	0.00 (−0.18, 0.17)	Ref.	0.172
*Gastrointestinal*								
Lack of appetite								
All	1175	**−0.05** (**−0.09**, **0.00**)	−0.01 (−0.06, 0.03)	−0.02 (−0.06, 0.03)	**−0.05** (**−0.10, −0.01**)	**−0.04** (**−0.08, 0.00**)	Ref.	0.166
Premenopausal	454	**−0.08** (**−0.14**, **−0.01)**	−0.04 (−0.10, 0.03)	−0.04 (−0.10, 0.03)	**−0.09** (**−0.16, −0.02**)	**−0.06** (**−0.13, 0.00**)	Ref.	0.064
Postmenopausal	721	−0.03 (−0.08, 0.03)	0.00 (−0.06, 0.06)	−0.01 (−0.07, 0.05)	−0.02 (−0.08, 0.04)	**−0.03** (**−0.08, 0.03**)	Ref.	0.700
Diarrhoea								
All	1169	**0.16** (**0.04**, **0.27**)	**0.15** (**0.03**, **0.27**)	**0.17** (**0.05**, **0.29**)	**0.15** (**0.03**, **0.27**)	**0.18** (**0.06**, **0.30**)	Ref.	**0.005**
Premenopausal	451	0.21 (−0.01, 0.42)	0.16 (−0.04, 0.37)	0.14 (−0.07, 0.35)	0.19 (−0.02, 0.41)	0.19 (−0.02, 0.40)	Ref.	0.066
Postmenopausal	718	0.13 (−0.01, 0.27)	0.14 (0.00, 0.29)	**0.19** (**0.04**, **0.33**)	0.12 (−0.03, 0.26)	**0.17** (**0.03**, **0.32**)	Ref.	**0.036**
*Psychological*								
Feeling nervous								
All	1166	**0.13** (**0.02, 0.23)**	0.06 (−0.05, 0.16)	0.09 (−0.01, 0.2)	0.06 (−0.05, 0.16)	**0.16** (**0.06, 0.26**)	Ref.	0.110
Premenopausal	454	**0.17** (**0.00, 0.34**)	0.13 (−0.04, 0.29)	0.16 (0.00, 0.33)	**0.19** (**0.02, 0.36**)	**0.18** (**0.01, 0.34**)	Ref.	**0.040**
Postmenopausal	721	0.09 (−0.04, 0.22)	0.01 (−0.12, 0.15)	0.05 (−0.08, 0.18)	−0.03 (−0.16, 0.11)	**0.15** (**0.02, 0.28**)	Ref.	0.710

^a^Difference in mean change comparing 0 to 20 mg (ref.) tamoxifen at end of treatment (at 6 months or date of discontinuation).

^b^*P* values for linear association between dose and symptom severity change.

Significant values are in bold (*P* < 0.05).

### Comparing low dose vs high dose

**Fig. 2:**
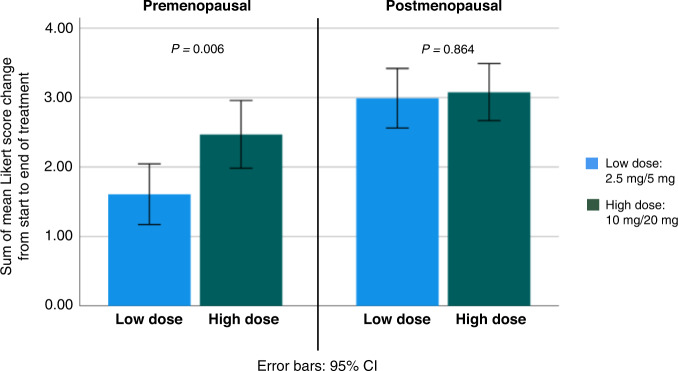
Symptom severity change by low (2.5 mg, 5 mg) and high (10 mg, 20 mg) dose tamoxifen and stratified by menopausal status. Sum of mean Likert score change is calculated from start to end of treatment, in symptoms significantly related to tamoxifen exposure regardless of menopausal status (hot flashes, cold sweats, night sweats, vaginal discharge, and muscle cramps). *P* value for difference between low and high dose within each menopausal group. Symptom severity change for each of the symptoms included can be found in Supplementary Fig. 1.

To contrast the effect of low-dose tamoxifen (2.5 and 5 mg) to high-dose (10 and 20 mg), we compared the sum of mean Likert score change (from baseline to end of treatment) in the five side effects (‘hot flashes’, ‘night sweats’, ‘cold sweats’, ‘vaginal discharge’ and ‘muscle cramps’) significantly associated with 20 mg tamoxifen in both premenopausal and postmenopausal women. We found a significant difference in the severity of side effects in premenopausal, but not in postmenopausal women. Premenopausal women at low-dose tamoxifen reported 34% less severity of side effects compared to women in the high-dose group (sum of mean Likert score change: 1.61 (95% CI: 1.17–2.04) versus 2.47 (95% CI: 1.98–2.96)) (Fig. 2).

When comparing the proportion of women increasing in Likert score it could be seen that significantly fewer premenopausal women in the low-dose arm reported an increase in Likert score, regardless of the difference in score, compared to women randomised to 10 and 20 mg of tamoxifen (Supplementary Fig. 2A). A finding not seen in postmenopausal women (Supplementary Fig. 2B).

## Discussion

In our comprehensive analyses of tamoxifen side effects in 1175 healthy women, five symptoms demonstrated an association with tamoxifen in both pre- and postmenopausal women. When comparing these side effects in women randomised to low-dose (2.5 and 5 mg) versus high-dose (10 and 20 mg) tamoxifen, the difference of severity from start to end of treatment was 34% lower in the low-dose group, a finding restricted to premenopausal women.

Few studies describe tamoxifen-induced side effects [14, 33, 34], or the effect of lower tamoxifen doses in healthy women. Most published studies include cancer patients with a plethora of treatments, making it hard to isolate the tamoxifen-specific side effects. In agreement with previous studies on tamoxifen side effects, including patients or healthy women, we found vasomotor symptoms such as hot flashes, cold- and night sweats, and gynaecological problems (vaginal discharge) to be strongly associated with tamoxifen. [2, 9, 17, 20, 26, 33–35] Muscle cramps had the largest mean severity change of all symptoms when contrasting placebo to 20 mg of tamoxifen. Muscle cramp is mentioned as common in the Summary of Product Characteristics (SmPC) of 20 mg tamoxifen [12], but sparsely in published papers and information targeted to patients [36, 37]. Skin reactions (skin rashes and itching) are poorly described in the literature, but in a systematic review by Andrew et al. 19% of tamoxifen users experienced cutaneous reactions [38].

In our study, 34 of the total 48 predefined symptoms were not significantly associated with tamoxifen. Ten questions covered psychological well-being whereof only ‘feeling nervous’ was associated with tamoxifen use, but unexpectedly with a negative direction. These findings contrast patient information, where mood swings and depression are described as common tamoxifen side effects [36, 37]. However, our findings are in agreement with other clinical trials including healthy women, where no association between tamoxifen use, distress, depression or emotional well-being were found [2, 9, 33, 39].

The clinical belief is that tamoxifen induces weight gain. The FACT-ES questionnaire only includes questions about weight gain and not about weight change or loss [40]. We found that premenopausal women on 20 mg of tamoxifen had a significant decrease in waist circumference, weight, and BMI, compared to the placebo group. Weight loss after tamoxifen is however supported by previous findings [41]. To our knowledge, this is the first study showing an effect of tamoxifen on waist circumference.

We found several symptoms strongly associated with menopausal status not previously described. The interaction of menopausal status on tamoxifen symptoms has not been studied extensively. In the NSABP P-1 trial, women of age 50–59 years reported more tamoxifen-associated symptoms compared to younger women. However, women <50 years reported the greatest relative increase in proportion of women reporting hot flashes at each examination during the study (36 months) [33].

Interestingly, we found that postmenopausal women discontinued treatment at nearly a twofold higher rate compared to premenopausal women. This finding contradicts earlier studies suggesting lower adherence in younger women [42]. However, the KARISMA trial was restricted to a 6-month treatment period and may not reflect adherence to long-term treatment. We did not find any significant differences in dropout rate when comparing low-dose versus high dose in pre- or postmenopausal women. Adherence to therapy has been demonstrated to be better in clinical trials than in a real-world setting [43, 44]; hence might the even distribution across dose arms of dropouts and compliance in this study reflect a ‘being a good participant-effect’. The two other known trials on low-dose tamoxifen, defined in both studies as 5 mg, did not include the clinically established 20 mg dose for comparison, thus making it difficult to compare their findings to our study [22, 32].

Importantly, we did not observe any reduction in severity of the top five symptoms when lowering the dose in postmenopausal women. The biological mechanisms behind the menopausal-dependent difference in tamoxifen side effects have not previously been addressed in detail. Three main factors probably influence the tamoxifen effect: tamoxifen metabolites, ER tissue expression, and hormone plasma concentrations. In the present study, there was no difference in tamoxifen metabolite (endoxifen) concentrations comparing pre- and postmenopausal women (data not published). We have previously shown that the expression of ER in the epithelial breast tissue of healthy women increases with increasing age [45, 46]. In a first biopsy-study nested within the KARISMA trial, we recently found that high-dose tamoxifen decreases the expression of epithelial ER and progesterone receptor (PR) expression in premenopausal, but not in postmenopausal women [47]. It could be that the age difference in ER expression reflects our findings of higher severity levels in postmenopausal women, also at lower doses.

From a clinical perspective, menopausal status is essential when assessing risk-benefit of the therapy of choice. In the U.S., tamoxifen is the only FDA-approved drug for breast cancer risk reduction among high-risk premenopausal women. However, high-risk postmenopausal women also have the option of raloxifene and aromatase inhibitors for preventive treatment.

The KARISMA trial has several strengths and weaknesses. Strengths include the fact that it is the only low-dose trial so far using the 20 mg standard dose as reference. Also, the double-blinded randomised study design with healthy participants, detailed information on side effects and high adherence with an overall compliance of 85.3%. We found few differences in baseline characteristics between treatment arms. With the exception of questions related to sexuality, the response rate for the remaining questions was close to 100%.

The major limitation of the study is that the treatment lasted for only six months. As the KARISMA trial was a dose-determination study, the trial was not designed for studying the long-term effects of tamoxifen. In a clinical setting, both risk-reducing and adjuvant tamoxifen therapy is recommended for at least five years [48–50]. Consequently, late-arising issues are not identified in this trial and need further investigation. Despite randomisation, differences at baseline were seen in hot flashes and smoking. Even so, the higher baseline score of hot flashes in the 20 mg reference arm would dilute, not strengthen, our results. Since this study only involved Swedish women attending the national screening programme results may not be generalisable to a more diverse population.

## Conclusion

The KARISMA study is the only randomised clinical trial specifically targeting side effects of both low and standard doses of tamoxifen in healthy women. This study demonstrates that low-dose tamoxifen substantially reduces the severity of the most prevalent side effects (hot flashes, night sweats, cold sweats, vaginal discharge and muscle cramps) in premenopausal women. We also show that menopausal status influences side effects of tamoxifen. If validated, these findings will have implications on future dosing strategies in the treatment of high-risk women as well as in adjuvant treatment. What dose of tamoxifen that provides optimal efficacy and tolerability needs to be further investigated.

## Supplementary information

Supplemental material

## Data Availability

The data underlying this article are available in the KARMA Research Platform at Karolinska Institutet, Sweden [karmastudy.org], and can be accessed up on request by contacting the corresponding author.
