# Supplementary material for: Side effects of low-dose tamoxifen: results from a six-armed randomised controlled trial in healthy women
Source: Br J Cancer. 2023 May 6;129(1):61–71. doi: 10.1038/s41416-023-02293-z (PMC10307785; doi:10.1038/s41416-023-02293-z)
Supplement: Supplementary file 1 — Supplemental material [file 41416_2023_2293_MOESM1_ESM.pdf]

Supplementary material

Side effects of low dose tamoxifen: Results from a six-armed randomized controlled trial in healthy women

Mattias Hammarström, Marike Gabrielson, Alessio Crippa, Andrea Discacciati, Martin Eklund, Cecilia Lundholm, Magnus Bäcklund, Yvonne Wengström, Signe Borgquist, Jenny Bergqvist, Mikael Eriksson, José Tapia, Kamila Czene, Per Hall

**Content**	Page
Supplementary Methods	2.
Supplementary Table 1	4.
Supplementary Table 2	6.
Supplementary Figure 1	7.
Supplementary Figure 2	8.

Supplementary Methods. KARISMA 48-item symptom questionnaire including origin of questions.

Question: Please mark your response for each question as it applies to the past 30 days.

	Source[a]	Not at all	A little bit	Some-what	Quite a bit	Very much	Don't know/ refuse
I have hot flashes	FACT ES	☐	☐	☐	☐	☐	☐
I have cold sweats	FACT ES	☐	☐	☐	☐	☐	☐
I have night sweats	FACT ES	☐	☐	☐	☐	☐	☐
I have vaginal discharge	FACT ES	☐	☐	☐	☐	☐	☐
I have vaginal itching/irritation	FACT ES	☐	☐	☐	☐	☐	☐
I have vaginal bleeding or spotting	FACT ES	☐	☐	☐	☐	☐	☐
I have vaginal dryness	FACT ES	☐	☐	☐	☐	☐	☐
I have pain or discomfort with intercourse	FACT ES	☐	☐	☐	☐	☐	☐
I have lost interest in sex	FACT ES	☐	☐	☐	☐	☐	☐
I have gained weight	FACT ES	☐	☐	☐	☐	☐	☐
I feel dizzy	FACT ES	☐	☐	☐	☐	☐	☐
I have been vomiting	FACT ES	☐	☐	☐	☐	☐	☐
I have diarrhea	FACT ES	☐	☐	☐	☐	☐	☐
I have headache	FACT ES	☐	☐	☐	☐	☐	☐
I feel bloated	FACT ES	☐	☐	☐	☐	☐	☐
I have sensitive/tender breasts	FACT ES	☐	☐	☐	☐	☐	☐
I have mood swings	FACT ES	☐	☐	☐	☐	☐	☐
I feel irritable	FACT ES	☐	☐	☐	☐	☐	☐
I have pain in my joints	FACT ES	☐	☐	☐	☐	☐	☐
I have muscle cramps	SMPC	☐	☐	☐	☐	☐	☐
I have skin rashes	SMPC	☐	☐	☐	☐	☐	☐
I have fragile mucous membranes	SMPC	☐	☐	☐	☐	☐	☐
I have sight/ eye changes	SMPC	☐	☐	☐	☐	☐	☐
I have abdominal obesity	KARISMA	☐	☐	☐	☐	☐	☐
I have heart palpitations	KARISMA	☐	☐	☐	☐	☐	☐

I have dry eyes	KARISMA	☐	☐	☐	☐	☐	☐

Question: Here is a list of symptoms. How much did the symptom bother or distress you during the past 30 days.

		Not at all	A little bit	Some-what	Quite a bit	Very much	Don't know/ refuse
Difficulty concentrating	MSAS	☐	☐	☐	☐	☐	☐
Pain	MSAS	☐	☐	☐	☐	☐	☐
Lack of energy	MSAS	☐	☐	☐	☐	☐	☐
Cough	MSAS	☐	☐	☐	☐	☐	☐
Feeling nervous	MSAS	☐	☐	☐	☐	☐	☐
Dry mouth	MSAS	☐	☐	☐	☐	☐	☐
Nausea	MSAS	☐	☐	☐	☐	☐	☐
Feeling drowsy	MSAS	☐	☐	☐	☐	☐	☐
Numbness/tingling in hands/feet	MSAS	☐	☐	☐	☐	☐	☐
Difficulty sleeping	MSAS	☐	☐	☐	☐	☐	☐
Problems with urination	MSAS	☐	☐	☐	☐	☐	☐
Shortness of breath	MSAS	☐	☐	☐	☐	☐	☐
Feeling sad	MSAS	☐	☐	☐	☐	☐	☐
Worrying	MSAS	☐	☐	☐	☐	☐	☐
Itching	MSAS	☐	☐	☐	☐	☐	☐
Lack of appetite	MSAS	☐	☐	☐	☐	☐	☐
Difficulty swallowing	MSAS	☐	☐	☐	☐	☐	☐
Change in the way food tastes	MSAS	☐	☐	☐	☐	☐	☐
Hair loss	MSAS	☐	☐	☐	☐	☐	☐
Constipation	MSAS	☐	☐	☐	☐	☐	☐
Swelling of arms/legs	MSAS	☐	☐	☐	☐	☐	☐
"I don't look like myself"	MSAS	☐	☐	☐	☐	☐	☐

[a] Sources of origin:

- FACT ES= Functional Assessment of Cancer Therapy - Endocrine Symptoms, version 4, Additional concerns [24]
- MSAS= Memorial Symptom Assessment Scale [28]
- SMPC= Summary of Product Characteristics (Mylan 20 mg) [8]
- KARISMA= Identified through literature or anecdotally

Supplementary Table 1. Mean Likert-score at baseline in the five symptoms significantly related to tamoxifen exposure regardless of menopausal status, by dose and menopausal status.

		Tamoxifen dose in mg					
Characteristic	**All**	**0**	**1**	**2.5**	**5**	**10**	**20**
Number of women included	1175	202	193	194	188	199	199
Baseline symptom score	Mean (95% CI)	Mean (95% CI)	Mean (95% CI)	Mean (95% CI)	Mean (95% CI)	Mean (95% CI)	Mean (95% CI)
Hot flashes							
All (n:1167)	0.51 (0.46, 0.56)	0.46 (0.35, 0.56)	0.51 (0.51, 0.39)	0.42 (0.31, 0.53)	0.55 (0.42, 0.67)	0.44 (0.33, 0.56)	0.68 (0.55, 0.81)
Premenop. (n: 451)	0.27 (0.22, 0.33)	0.21 (0.08, 0.35)	0.31 (0.16, 0.46)	0.20 (0.05, 0.35)	0.19 (0.07, 0.32)	0.24 (0.12, 0.36)	0.47 (0.29, 0.65)
Postmenop. (n: 716)	0.66 (0.59, 0.72)	0.59 (0.44, 0.73)	0.65 (0.48, 0.81)	0.56 (0.40, 0.71)	0.77 (0.59, 0.94)	0.57 (0.41, 0.73)	0.82 (0.64, 1.00)
Cold sweats							
All (n:1170)	0.16 (0.14, 0.19)	0.16 (0.09, 0.22)	0.17 (0.10, 0.23)	0.13 (0.07, 018)	0.21 (0.13, 0.29)	0.16 (0.09, 0.23)	0.17 (0.10, 0.24)
Premenop. (n: 449)	0.12 (0.09, 0.16)	0.13 (0.03, 0.23)	0.13 (0.04, 0.21)	0.13 (0.05, 0.22)	0.13 (0.03, 0.22)	0.08 (0.02, 0.14)	0.14 (0.04, 0.25)
Postmenop. (n: 716)	0.19 (0.15, 0.23)	0.17 (0.08, 0.26)	0.20 (0.11, 0.29)	0.12 (0.04, 0.20)	0.27 (0.15, 0.38)	0.20 (0.10, 0.31)	0.18 (0.08, 0.29)
Night sweats							
All (n:1170)	0.65 (0.60, 0.70)	0.67 (0.55, 0.80)	0.63 (0.50, 0.75)	0.59 (0.47, 0.70)	0.60 (0.48, 0.72)	0.64 (0.52, 0.76)	0.78 (0.65, 0.91)
Premenop. (n: 452)	0.54 (0.47, 0.62)	0.52 (0.33, 0.72)	0.51 (0.35, 0.67)	0.43 (0.28, 0.57)	0.46 (0.26, 0.66)	0.54 (0.36, 0.72)	0.80 (0.58, 1.01)
Postmenop. (n: 718)	0.72 (0.65, 0.79)	0.75 (0.59, 0.91)	0.71 (0.53, 0.88)	0.69 (0.53, 0.86)	0.69 (0.53, 0.85)	0.70 (0.54, 0.86)	0.78 (0.61, 0.94)
Vaginal discharge							

All (n:1170)	0.49 (0.45, 0.53)	0.40 (0.30, 0.49)	0.51 (0.40, 0.61)	0.51 (0.40, 0.61)	0.51 (0.40, 0.61)	0.45 (0.35, 0.56)	0.57 (0.46, 0.67)
Premenop. (n: 451)	0.90 (0.82, 0.98)	0.76 (0.56, 0.96)	0.89 (0.69, 1.08)	1.03 (0.83, 1.22)	0.96 (0.76, 1.16)	0.85 (0.65, 1.05)	0.94 (0.74, 1.13)
Postmenop. (n: 719)	0.23 (0.19, 0.26)	0.21 (0.12, 0.29)	0.23 (0.15, 0.32)	0.17 (0.09, 0.25)	0.22 (0.13, 0.32)	0.21 (0.12, 0.30)	0.33 (0.23, 0.42)
Muscle cramps							
All (n:1173)	0.45 (0.41, 0.50)	0.49 (0.38, 0.59)	0.48 (0.37, 0.60)	0.44 (0.32, 0.55)	0.42 (0.31, 0.53)	0.40 (0.30, 0.51)	0.48 (0.36, 0.59)
Premenop. (n: 453)	0.26 (0.20, 0.30)	0.20 (0.10, 0.30)	0.24 (0.11, 0.36)	0.26 (0.13, 0.39)	0.21 (0.06, 0.36)	0.17 (0.08, 0.26)	0.44 (0.25, 0.64)
Postmenop. (n: 720)	0.57 (0.51, 0.64)	0.64 (0.50, 0.78)	0.66 (0.49, 0.83)	0.55 (0.38. 0.72)	0.55 (0.39, 0.71)	0.54 (0.39, 0.70)	0.50 (0.35, 0.65)

Abbreviations: CI= Confidence interval

Supplementary Table 2. Mean individual change of three anthropometric measures, from start to end of treatment, comparing all doses including placebo to the 20 mg tamoxifen arm, for all women combined and stratified by menopausal status.

**Anthropometrics**	N	**Tamoxifen dose in mg**						
		**0** Mean change (95% CI) [a]	**1** Mean change (95% CI) [a]	**2.5** Mean change (95% CI) [a]	**5** Mean change (95% CI) [a]	**10** Mean change (95% CI) [a]	**20** Mean change	**Association with dose** p-value [b]
BMI (kg/m^2)								
All	1135	0.22 (0.00, 0.44)	0.15 (-0.08, 0.37)	**0.41 (0.18, 0.63)**	0.19 (-0.04, 0.42)	0.05 (-0.17, 0.27)	Ref.	**0.004**
Premenop.	436	**0.28 (0.01, 0.55)**	0.26 (0, 0.53)	**0.42 (0.16, 0.69)**	**0.35 (0.08, 0.62)**	**0.33 (0.06, 0.59)**	Ref.	**0.010**
Postmenop.	699	0.17 (-0.15, 0.48)	0.08 (-0.25, 0.40)	**0.40 (0.08, 0.72)**	0.09 (-0.23, 0.42)	-0.12 (-0.44, 0.20)	Ref.	0.062
Weight (kg)								
All	1135	0.59 (-0.02, 1.19)	0.38 (-0.22, 0.99)	**1.06 (0.46, 1.67)**	0.51 (-0.10, 1.13)	0.12 (-0.48, 0.72)	Ref.	**0.005**
Premenop.	436	**0.80 (0.04, 1.56)**	0.73 (-0.01, 1.47)	**1.15 (0.4, 1.89)**	**0.96 (0.21, 1.72)**	**0.87 (0.12, 1.61)**	Ref.	**0.009**
Postmenop.	699	0.43 (-0.42, 1.27)	0.18 (-0.70, 1.05)	**1.02 (0.15, 1.88)**	0.24 (-0.64, 1.12)	-0.34 (-1.20, 0.51)	Ref.	0.077
Waist circumference (cm)								
All	1130	0.74 (-0.81, 2.29)	-0.28 (-1.85, 1.29)	0.35 (-1.21, 1.91)	-0.61 (-2.19, 0.98)	-0.91 (-2.46, 0.63)	Ref.	0.454
Premenop.	433	**3.53 (0.88, 6.17)**	0.19 (-2.40, 2.78)	**2.63 (0.03, 5.24)**	0.73 (-1.90, 3.36)	1.80 (-0.78, 4.38)	Ref.	0.083
Postmenop.	697	-0.94 (-2.84, 0.96)	-0.50 (-2.45, 1.45)	-1.08 (-3.00, 0.85)	-1.42 (-3.38, 0.55)	**-2.61 (-4.52, -0.69)**	Ref.	0.623

[a] = Difference in mean change comparing 0 to 20 mg (ref.) tamoxifen at end of treatment (at six months or date of discontinuation)

[b] = P-values for linear association between dose and symptom severity change

Significant values in bold ($p < 0.05$)

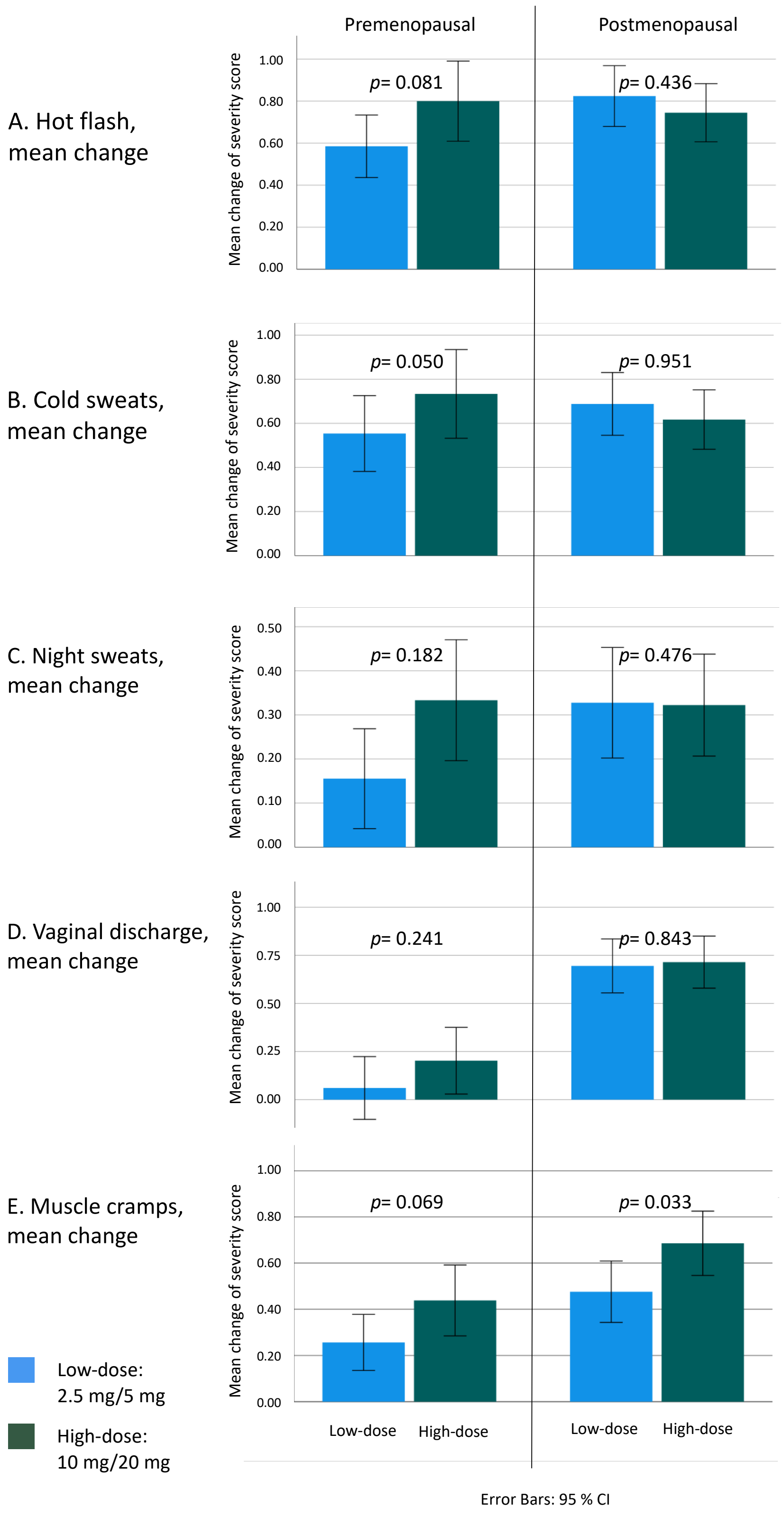

Supplementary Figure 1. Symptom severity change by low (2.5 mg, 5 mg) and high (10 mg, 20 mg) dose tamoxifen and stratified by menopausal status. Mean change of severity score is calculated from start to end of treatment. P-value for difference between low and high dose within each menopausal group.

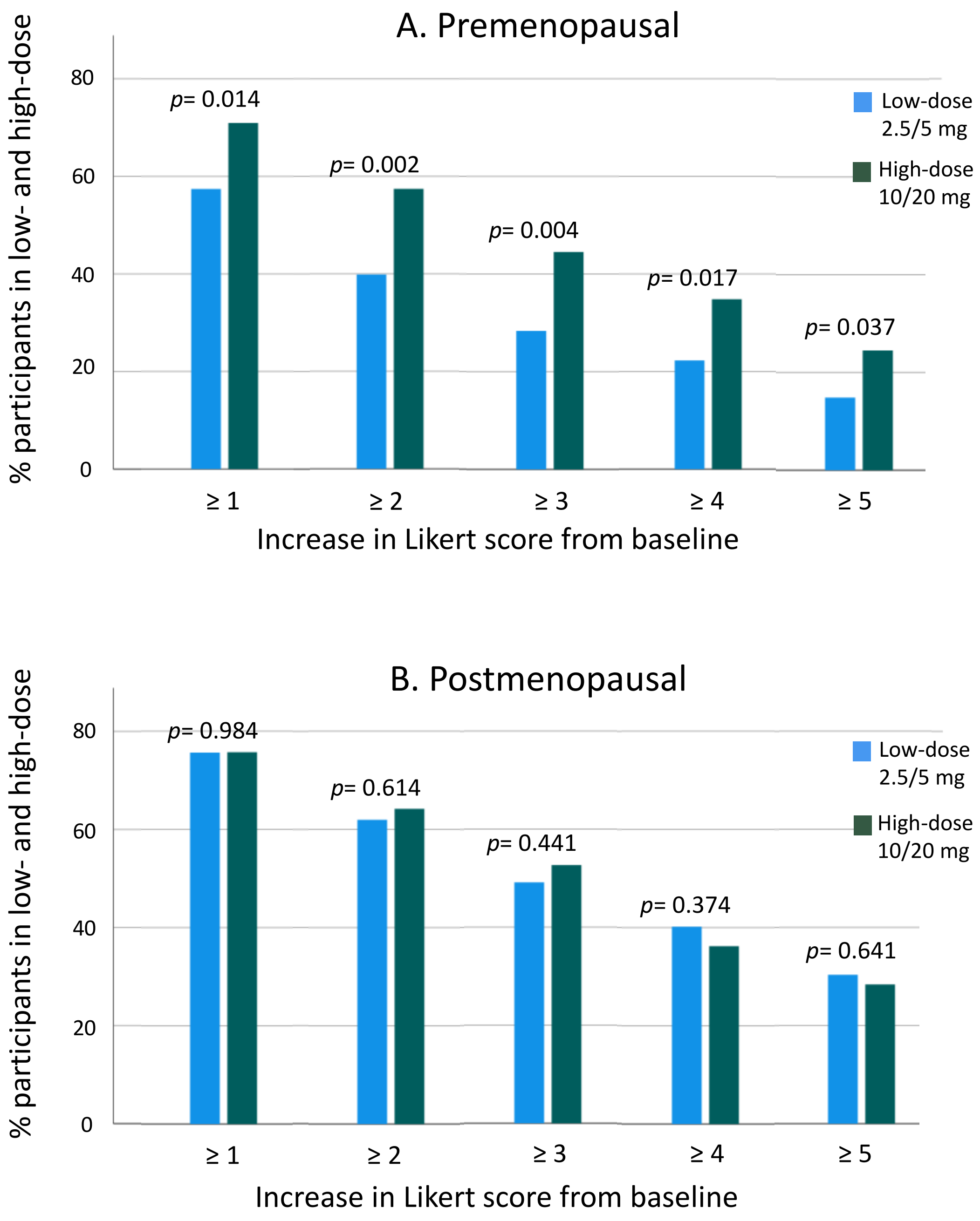

Supplementary Figure 2. Proportion of women increasing by ≥1, 2, 3, 4 and 5 in Likert score, comparing baseline to end of treatment in the five symptoms significantly related to tamoxifen exposure (hot flashes, cold sweats, night sweats, vaginal discharge, and muscle cramps) and stratifying by menopausal status and dose. P-value for test of difference between low and high dose.
